# Predicting the Annual Funding for Public Hospitals with Regression Analysis on Hospital’s Operating Costs: Evidence from the Greek Public Sector

**DOI:** 10.3390/healthcare10091634

**Published:** 2022-08-27

**Authors:** Paraskevi N. Zaza, Pantelis G. Bagos

**Affiliations:** Department of Computer Science and Biomedical Informatics, University of Thessaly, 35131 Lamia, Greece

**Keywords:** hospitals, health policy, health economics, operational costs, regression analysis, longitudinal data

## Abstract

The funding of public hospitals is an issue that has been of great concern to health systems in the past decades. Public hospitals are owned and fully funded by the government, providing in most countries medical care to patients free of charge, covering expenses and wages by government reimbursement. Several studies in different countries have attempted to investigate the potential role and contribution of hospital and clinical data to their overall financial requirements. Many of them have suggested the necessity of implementing DRGs (Diagnosis Related Groups) and activity-based funding, whereas others identify flaws and difficulties with these methods. What was attempted in this study is to find an alternative way of estimating the necessary fundings for public hospitals, regardless the case mix managed by each of them, based on their characteristics (size, specialty, location, intensive care units, number of employees, etc.) and its annual output (patients, days of hospitalization, number of surgeries, laboratory tests, etc.). We used financial and operational data from 121 public hospitals in Greece for a 2-years period (2018–2019) and evaluated with regression analysis the contribution of descriptive and operational data in the total operational cost. Since we had repeated measures from the same hospitals over the years, we used methods suitable for longitudinal data analysis and developed a model for calculating annual operational costs with an R²≈0.95. The main conclusion is that the type of hospital in combination with the number of beds, the existence of an intensive care unit, the number of employees, the total number of inpatients, their days of hospitalization and the total number of laboratory tests are the key factors that determine the hospital’s operating costs. The significant implication of this model that emerged from this study is its potential to form the basis for a national system of economic evaluation of public hospitals and allocation of national resources for public health.

## 1. Introduction

In 2015, all member states of the United Nations adopted 17 Goals for Sustainable Development to take action to end poverty, protect the planet and improve the lives and prospects of everyone, everywhere [1]. Sustainable Development Goal 3 seeks to ensure healthy lives and promote well-being for all at all ages. Health financing is critical for reaching the universal health coverage (UHC) they need without suffering financial hardship. Most countries from all over the world attempting to achieve this goal try to strengthen health financing, witnessing at the same time a stable annual increase of health expenditure. Globally, from 2000–2015, the average annual growth rate of healthcare spending was 4.0%; however, the average annual growth rate of global economy was 2.8% [2]. Through this process, cost containment and the fair allocation of public money to hospitals became necessary for the sustainability of both state economies and the public health structures themselves.

What has been attempted and increasingly adopted by the various health systems is the costing of medical services in hospitals based on the International Classification of Diseases ICD10, the usual practices–protocols for their management by the hospitals of each country, the prices of the purchase of medicines, sanitary material, and other materials, services necessary for the operation of health units and the average compensation of employees. Diagnosis related groups (DRGs) were developed through this procedure, under which hospitals are reimbursed with specific amounts based on their activity rather than on their pre-existing costs. However, there is growing frustration with their implementation and in particular their unintended consequences [3]. Firstly, it is uncertain if this type of funding in fact improves activity and efficiency of hospital services. Secondly, activity-based funding may adversely affect the equity of healthcare systems as the funding provided by a specific DRG value is fixed. There is a potential incentive for hospitals to give preference to patients without special needs or complex illness trajectories. Thirdly, as hospitals are rewarded for activity, it is a common concern that activity-based funding may motivate hospitals to focus on maximizing earnings rather than offering integrated care and the highest quality of treatment [4]. Public hospitals are nonprofit organizations (NPOs) and should not seek profit, but instead should seek to contribute to society at large. However, in recent years, there has been observed upcoding by DRG-funded hospitals costing a substantial amount of money in many developed countries [5,6,7]. For this reason, efforts are being made to develop tools and techniques to detect upcoding [5,8]. Evidence also suggests substantial increases in admissions to post-acute care following hospitalization, with implications for system capacity and equitable access to care [9]. Meta-analysis of 18 studies reveals that DRGs-based payment may have cost-saving implications by lowering length of stay (LOS), whereas hardly reduce the readmission rates. Policymakers considering adopting DRGs-based payments should pay more attention to the hospital readmission rates compared with cost-based payment [10]. To implement activity-based funding, health care leaders have emphasized the complexity of the process, which requires the following: organizational commitment; adequate infrastructure; human, financial and information technology resources; change champions and a personal commitment to quality care [11].

From January 2012, Greece adopted for the first time a DRG system [12], which was derived from the analysis and combination of medical practice coding systems from other countries [13]. The process was quite difficult and demanding in terms of changes that had to be done in the hospitals’ information systems and the employees involved (health and administrative staff) who needed training from both hospitals and the main insurance provider (NOPHS) National Organization for the Provision of Health Services that had to check the hospitals’ invoices with their claims. Very often, after reviewing the invoices submitted by hospitals to NOPHS, which in most cases lacked the necessary supporting documents, there was a discrepancy between the hospitals’ claims and the final amounts approved by NOPHS for settlement. This caused very long delays in the clearance and reimbursement of hospitals, which in turn caused them financial strangulation. To address this problem, the Ministry of Health for several years directly funded the hospitals each year from the state budget with the 50% of their annual expenses and at the same time erased from NOPHS claims equal to the subsidies they had received [14,15]. NOPHS, on the other hand, every year paid back the hospitals only a part of their claims, because of the reasons mentioned above. All this, progressively, resulted in the determination of the annual budget not taking into account the requirements of the hospitals based on the Greek DRGs, but the expenses of previous years adjusted each time with the available amounts of the annual state budget that were determined for the coverage of health care expenditures. Although Greece is currently making efforts to upgrade both the DRGs and the procedures required for their integration and implementation by public hospitals, what is currently happening is that they are being funded based on a longitudinal approach, without any real evaluation of the work achieved by each health unit. For this reason, there are large variations in average costs per patient, per bed and per day of hospitalization between different health regions or even between hospitals of the same category. In addition to all this, the economic crisis that Greece has faced in recent years has caused budget deficits, which in turn have led to shortages of materials or inability to pay for services necessary for the safe operation of hospitals. In any case, whether we were dealing with hospitals that receive more supportive fundings or those that were more underprivileged, one thing is common to all of them, the effort to operate safely for all patients regardless of the diseases treated or the days of hospitalization needed for treatment. Therefore, the trend will certainly be obvious and should be investigated.

In this context and with the help of available operational and financial data from the Ministry of Health from several years, we analyzed the operating costs of all public hospitals in Greece, of a 2-year period (2018–2019) to identify those factors that have a statistically significant impact on the individual or total operating costs of hospitals. The specific years were chosen because they were years with normal operating conditions, in contrast to those of 2020 and 2021 in which hospital operations in all over the world were significantly changed due to COVID-19. In addition, they were the years with the most reliable data on the platform from which we obtained the information needed, as the uniform coding of hospital expenditure had recently been completed and adopted by all hospitals. The goal was to find a simple statistical model for estimating the annual operating costs, and thus the necessary funding of each hospital based on its output (patients, surgery, days of hospitalization, laboratory tests, etc.) or its characteristics (size, location, category, specialty, etc.).

## 2. Materials and Methods

Our main data source was “Business Intelligence System” (BI-Health) an online platform of the Ministry of Health, in which, every month, Greece’s public hospitals enter mandatory, operational and financial data [16]. The “BI-Health” plays a central role in the organizational, operational, and financial modernization of the National Health System of Greece. It ensures the collection and process of detailed and aggregated data of the State’s Public Hospitals, at a central operational level and allows the dissemination of information to the management mechanisms with the goal of improving the quality of health services provided.

In many cases, it was necessary to cross-reference the data contained in the BI-Health, and for this, information from the official websites of the hospitals was used. Additionally, due to the adoption of the common codification of costs in Greek hospitals per category of material or service that was completed by the end of 2018, many entries of different costs were noticed in the same cells of BI-Health, which made it difficult to separately identify each category. For this reason, we searched the relevant uploads of each hospital on the Transparency Program [17]. Since 2010, all government institutions have been obliged to upload their acts and decisions onto the internet, with special attention to issues of national security and sensitive personal data. Each document is digitally signed and assigned a unique internet uploading number (IUN), certifying that the decision has been uploaded at the “Transparency Portal”. Thus, we were able to identify information that was not visible or not registered on the BI platform.

Individual-hospital characteristics that were available on the BI platform were screened to be included in our multivariable models as independent variables, to assess their association with the total operating cost. The hospital’s type is the first characteristic that reflects major information about the size of each one, the number and the complexity of the cases it handles. We have categorized 5 types of Greek public Hospitals: Small Hospitals/Health Centers (bed capacity up to 100), General Hospitals, University Hospitals and Specialized Hospitals of two types (cancer (type II) and other specialties (type I)). The number of beds was also included in our model, as it indicates its size and its potential number of hospitalized patients. The area in which each hospital is located is also recorded because of the potential difficulties that may arise, and therefore greater financial requirements, due to long distances from companies providing materials or services necessary for their operation. The separation recorded concerned the location of each hospital, in mainland or island Greece. Τhe existence or not of special units such as the Intensive Care Unit, Increased Care Unit and Artificial Kidney Unit, was information that was included as well, because of the possible additional management and operating costs. The number of employees, that is, permanent staff and auxiliary staff, was also examined as it indicates the hospitals size and the number of cases it can serve.

Τhe total annual healthcare activities performed in each hospital were also recorded and evaluated for their association with operating costs: the annual number of patients, internal and external. The annual number of internal patients in special clinics. The number of laboratory tests (bio-pathological tests, endoscopic examinations and invasive diagnostic tests), medical imaging tests and others, as a whole and individually were recorded for both years. The annual number of hemodialyses performed in the artificial kidney units and finally the total number of surgeries (both urgent and scheduled) were also examined for a possible association with operational costs.

The total operating costs of Greek public hospitals are divided into individual costs of various categories. The BI platform records separately the costs for the purchase of drugs, sanitary materials, reagents and other chemical materials, prosthetic organs, orthopedic materials, blood donation products, radiological materials, other non-medical equipment, services and the salaries of auxiliary staff. The salaries of permanent hospital staff in Greece are covered by the regular state budget. The category of services in the BI platform includes the energy costs of hospitals, i.e., electricity and costs for heating the facilities. These costs were excluded from this study because they are data that were examined separately, as they are more influenced by the characteristics of the hospitals than by the total number of cases they manage. This has been researched and demonstrated in a previous study [18]. The salary costs of hospital auxiliary staff, although included in the annual budget and managed by the hospitals each year, were excluded from this study as well, as they are perfectly measurable based on the number of people who need to be recruited as auxiliaries and the total number of staff participating in the hospital’s on-call schedule. The other cost categories were analyzed separately and combined. The amounts analyzed represented almost 87% of the hospitals’ total operating costs.

The regression analysis method has been used to predict health care costs based on demographic and descriptive patient data, combined with corresponding health care costs from earlier years, obtained from insurance providers [19,20,21,22], in several countries. In this study, the available data allowed us to perform regression analysis on the operating costs of hospitals in relation to the characteristics mentioned above. The operating costs that were our dependent variables deviated significantly from the normal distribution, so we transformed them and used their logarithms for the analysis applying, subsequently, multiple linear regression models. Since we had repeated measures from the same hospitals over two years, we first separately analyzed the data for each year and then we used methods suitable for longitudinal data analysis [23]. We applied and compared several models. First, we used ordinary least squares (OLS) with the Huber/White (Sandwich) estimator of variance [24,25,26]. In order to increase the efficiency of estimation, we applied the seemingly unrelated regression (SUR) models [27], for the individual hospital costs, which computes simultaneous but separate regression models for each of them, assuming that the (contemporaneous) errors associated with the dependent variables may be correlated. We also conducted a multivariate regression analysis, as there was evidence of large correlations between the dependent variables, which were the individual hospital costs. For the total cost analysis from both years, we used OLS regression model clustering our data by hospital and random effects regression analysis configuring our data appropriately for this analysis, declaring the “Hospital” as the panel variable and “Year” as the time variable. The Breusch and Pagan Lagrangian multiplier test for random effects was used to test the appropriateness of the random effects model [28,29,30]. Akaike’s information criterion (AIC) and Bayesian information criterion (BIC) were used to compare competing models. All analyses were performed with Stata version 13 [31] using the commands: reg, sureg, mvreg, and xtreg. In all cases, significant results were considered those with a *p*-value < 0.05.

## 3. Results

Greece is divided in seven Health Districts governing, in cooperation and under the guidance of the Ministry of Health, 125 public hospitals of different sizes and specialties scattered throughout the mainland and island country. Some of the hospitals have a common administration and a common recording of their financial data on the BI platform, which is why they were considered as one in our research. We categorized Greece’s public hospitals in five categories (small hospitals/health centers, general hospitals, university hospitals and specialized hospitals). From the 125 hospitals that exist in the country (mainland and the islands), 121 were included in our sample. Two very small hospitals were completely excluded due to the insufficient data they provided, whereas two others were recorded as one with their interconnected hospitals. The distribution of the hospitals according to their type, the area in which they are located, and their number of beds are shown in Table 1.

Small hospitals have a range of 20 to 80 beds, they provide basic medical services and perform simple surgical procedures and in most cases, they are located in isolated places, i.e., islands or in remote mountain areas. General hospitals have between 61 and 945 beds and have the majority of medical specialties providing corresponding services. University hospitals are those that are linked to a medical school and usually they provide more specialized services, dealing with more difficult cases. Their own range of beds is from 368 to 863. In the category of Specialized Type I hospitals we have included psychiatric hospitals, gynecological, ophthalmological, pediatric and some rehabilitation hospitals. Finally, type II specialized hospitals are cancer hospitals with a bed capacity from 107 to 380.

Initially, and in order to confirm the aforementioned difference between actual expenditure and the corresponding reimbursements claimed by hospitals from ΝOPHS, we conducted a Wilcoxon signed-rank test to check the equality between these two figures from both years. The audit confirmed the existence of statistically significant differences between the actual costs and the corresponding hospital reimbursements based on the Greek DRGs, even before the final NOPHS audit of the latter. In particular, it showed that hospitals tend to report services that are billed more expensively than they actually cost them.

As mentioned before, our research focused on actual costs and the factors that influence them. So, firstly we conducted a regression analysis for each cost separately. Our dependent variables were the annual costs for drugs, sanitary materials, chemical reagents, prosthetic organs, orthopedic materials, radiological materials, other non-medical equipment and services. Our independent variables were all those containing the total work produced by hospitals per year and those with information on hospital characteristics. The independent variables had high pairwise correlations between them (Table 2). As described in the Materials and Methods section we conducted regression analysis for each of the different costs using three different methods (OLS with the Sandwich variance estimator, multivariate regression analysis and SUR). All analysis were conducted for each year’s data separately and from both years, categorizing them by hospital.

**Figure 1:**
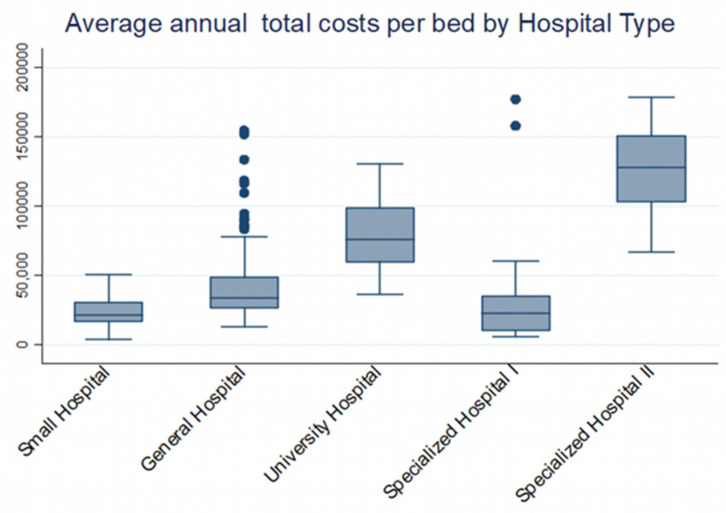
Average annual total cost (€) per bed for all types of hospitals.

Using these methods we arrived at models with R² (Coefficient of determination) ranging from 33% to 90% depending on the category of material for which the cost was being examined. Models from costs for materials or categories of products or services, that existed in all hospitals had a better fit to our data with an R² of over 85%. Those relating to materials used by a few hospitals either due to specialization or due to modernization of procedures (such as radiology material-films) had models with lower R². The key common element of the models obtained with all three regression methods was the almost identical independent variables that had statistically significant effects on the individual hospital costs that were our dependent variables. In addition, and given that it is impractical to use different models to estimate the costs of each category separately and then compute the total operational cost, we proceeded to a regression analysis for the total hospital operating cost as our dependent variable. Likewise, this analysis was performed for each year (2018 and 2019) and in total. The Breusch and Pagan test provided evidence of significant differences across hospitals (Figure 1).

So, we conducted a linear regression, clustering our data by hospital, and next we conducted regression for the panel data with a random effects model. We arrived at models with an R² slightly greater than 95% (Table 3).

The mathematical formulas that express our models are the following:

- The OLS Linear’s Regression model:

Log(AllCosts) = 12.02422 + 0.0172 × Beds + β_2_ × HospitalType + β_3_ × HospitalType#Beds + β_4_ × Beds#Days + β_5_ × ICU + 0.0000241 × InPatients + 0.0001878 × Days + 0.001168 × Employees + β_9_ × Location + β_10_ × HospitalType#Days

- The Random Effect Model:

Log(AllCosts) = 12.1828 + 0.0273 × Beds + β_2_ × HospitalType + β_3_ × HospitalType#Beds + β_4_ × Beds#Days + β_5_ × ICU + 0.0000184 × InPatients + 0.000003 × Days + 0.0000002 × LabTests + 0.0006503 × Employees

* The values of the coefficients β_2_, β_3_, β_4_, β_5_, β_9_ & β_10_ for the indicator variables in the above models are given in Table 3 for each case individually.

Although the differences between the two models are very small in terms of the coefficients of their common independent variables, calculating the values of the Akaike’s information criterion and Bayesian information criterion for the two models, we conclude that the most appropriate model for our data is the random effects model (Table 4).

Based on the random effects model, the variables that appear to have a statistically significant effect on the total costs of hospitals are the number of beds, the type of hospital, the existence of an intensive care unit, the number of employees (variables that relate to hospital characteristics) and from the variables that indicate work output are, the total annual inpatients, the days of hospitalization, but with a different weight for different number of beds, the total laboratory tests performed and the total number of employees.

**Figure 2:**
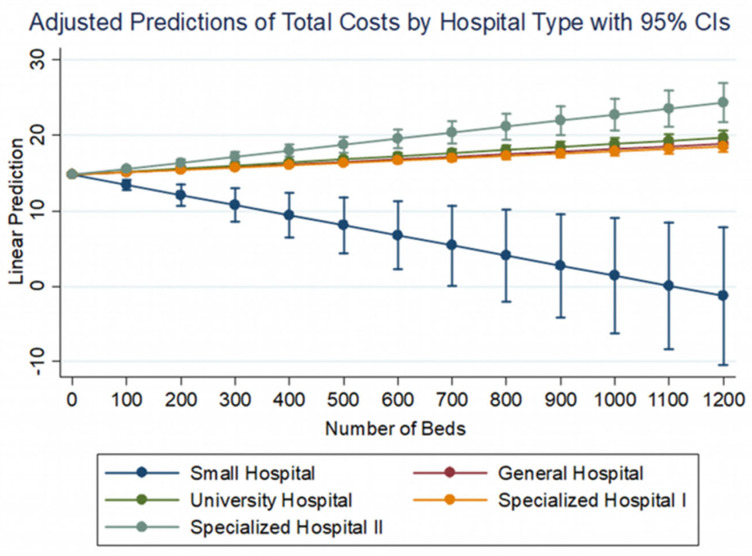
The impact of extra beds on total hospital costs for each type of hospital.

In particular, the number of beds have a positive effect on total costs with a different weight for each type of hospital (Figure 2). It is obvious that only in small hospitals the increase in beds leads to a reduction in costs, achieving economies of scale, which is perfectly logical and expected as the number of beds and patients handled by these hospitals is quite small, while the majority of small hospitals are located in remote areas, which makes them more costly.

**Figure 3:**
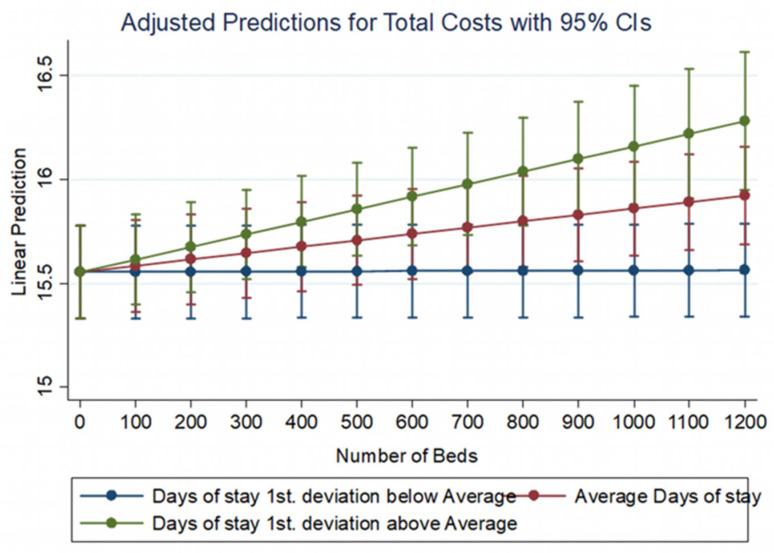
The impact of hospitalization days on total costs for different numbers of beds.

Hospitalization days also have a positive effect on total costs with different intensity depending on the number of beds in a hospital (Figure 3). If inpatient days remain low, i.e., below the average number of hospitalization days, then the effect of their increase on total operating costs is very small or zero regardless of the number of beds in the hospital. In other cases where inpatient days are close to or above average, the impact of their increase on total costs is progressively greater as the number of beds increases. Both models show a positive effect of inpatients on costs and the number of laboratory tests performed in each hospital. The existence of an intensive care unit also adds an extra amount to the operating costs for each hospital.

The same is observed with the location where each structure is located, which, as can be seen from the simple linear regression model when it is an island, means the costs are higher. This is completely expected, since the island country, due to distances and modes of transportation to mainland Greece, requires more expensive shipping costs for products or services necessary for the operation of hospitals.

The number of employees of each hospital has an increasing impact on operating costs, as it is an indication of both its size and the number of patients it can serve.

**Figure 4:**
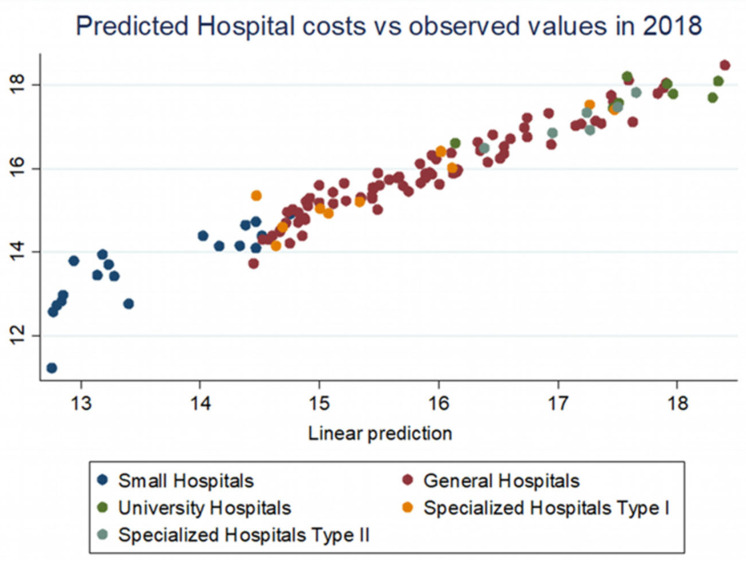
Predicted hospital costs vs. observed values in 2018.

**Figure 5:**
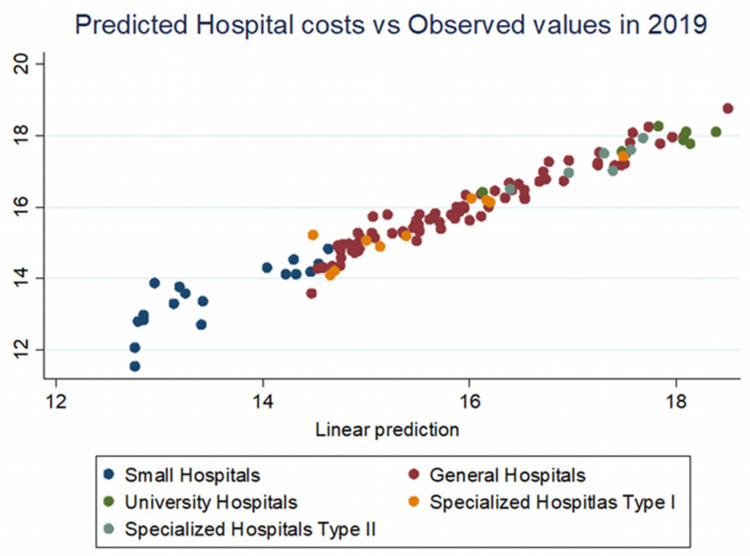
Predicted hospital costs vs. observed values in 2019.

By making the projections of total expenditure based on the multiple linear regression model, using the two year’s data, we find the adjustments need to be done to the operating costs and thus to the hospitals’ funding’s, either upwards or downwards (Figure 4 and Figure 5). Considering the hospital’s characteristics and the range of services it provided, regardless of their type or complexity, it is possible to assess its operation in terms of costs and identify the interventions necessary to provide a safer and better-quality service for patients. Regardless of whether the regression model was derived from sufficient or insufficient budgets of those years, the trend is distinct and measurable. Depending on each year’s available state’s budget resources for hospital operations, the Ministry of Health can intervene in funding in a fair manner and within safe limits.

## 4. Discussion

The need for continuous improvement of health care systems is undeniable. The way in which public hospitals are financed plays a very important role in their efficient and safe operation and, by extension, in the services that patients receive. Activity-based funding under the DRG system has been linked to various impacts on both the quality of services provided [9,10,32] and the finances of insurance providers, both governmental and non-governmental, that reimburse hospitals for health services [5,8,32,33]. In addition, implementing activity-based reimbursement reforms for a country’s public hospitals is an incredibly complex process requiring the following: organizational commitment; adequate infrastructure; human, financial and information technology resources; change champions and a personal commitment to quality care [11]. The model presented in this study is simple but powerful enough to form the basis for a National System of Financial Evaluation of public hospitals and allocation of national public health resources. Improving the records and constant feeding back with new data can continuously optimize the model, thus achieving the identification of those parameters that affect costs. The detailed segmentation of total costs into individual costs, for hospitals where large deviations of estimates from actual costs arise, can highlight waste or even good practices. Accomplishing savings in order to reduce per capita costs need not and should not be achieved by reductions in the quality of services provided and the days of hospitalization of patients, which in the end costs hospitals more due to an increase in complications or readmissions. Instead, the following practices can be adopted to reduce costs in a public hospital:

- Centralization of procurement of commonly used drugs and materials.
- Implementation of disease and materials management protocols.
- Continuous monitoring of consumables and inventories.
- Use of renewable energy sources.
- Maintenance and upgrading of old and high-cost medical and mechanical equipment, etc.

The main difficulties of this practice, for any health system willing to adopt it, are related to the collection of a large volume of operational and descriptive data from hospitals of all sizes and specialties while monitoring their costs in order to arrive at those variables that have a statistically significant impact on the operating costs through regression analysis. In this study, statistical models were obtained based on hospital expenditures implemented by the country’s hospitals based on their respective state funding in 2018 and 2019, which involves a risk of bias. However, regardless of whether or not these two years’ fundings were correct and adequate for each hospital, what is of interest is the trend for safe operation of each hospital. This means a certain amount of spendings on drugs and materials and a certain amount of operating costs for maintenance and services depending on the size of the hospital, its staff, its specialty, or its location and, of course, in conjunction with its output (patients, hospital days, laboratory tests). In addition, the bias error from using financial data from years when there were inadequate budgets can be reduced by using data from more years.

## 5. Conclusions

The triple aim approach for improving new healthcare systems, recommended in 2008 by the American Institute for Healthcare Improvement (IHI [34]), aims simultaneously to: improve care, improve the health of populations and reduce the per capita costs of health care. All three concepts are fully interrelated, and each can have a positive or negative impact on the other. The service and treatment of individual diseases determines both the levels of care and the improvement or otherwise of the health of the population. However, linking it to hospital reimbursements and thus to the reduction of per capita expenditure may have undesirable effects on the overall common objectives.

Approaching hospital reimbursements from the point of view of the individual hospital’s characteristics and their overall work, free from the type of cases it served, priced at the rates of a dynamic and highly volatile global market, to which all public hospitals can now turn, can lead to safer estimates. The hospital’s type, its number of beds and employees, the existence of an Intensive Care Unit and its location are information that is very easy to obtain. The number of patients and days of hospitalization are also data that are easily tracked by all hospital information systems. This study has proved that these variables contain all the information that are responsible for changes in the operating costs of public hospitals in Greece.

The use of this practice by other countries, to identify those factors that in their health system affect the overall costs regardless of the type of cases served in each public health facility is worth exploring, especially from countries that have not yet implemented a billing system or are not foreseen to implement one in the near future, due to lack of health protocols for the management of each disease, sufficient staff or other prerequisites for the implementation and use of a DRG billing and reimbursement system. Additionally, since all public health structures serve all diseases, according to their dynamics and their specialty, it would be worth exploring this practice as an alternative way of calculating the necessary funding of public hospitals, free of any link between the illness or the duration of hospitalization, even of health personnel with economic data, thus minimizing the undesirable side effects of such interconnections. On the other hand, the difficulties and biases that may arise in the design of models for estimating the necessary financing of public hospitals, using the practice proposed by this study, are minor in relation to the adoption of any kind of costing method for hospital services, and can be easily overcome, as mentioned above.

## Figures and Tables

**Table 1:** Hospital distribution in Greece per health district and hospital type.

Health District	Small Hospitals	General Hospitals	University Hospitals	Specialized type I Hospitals	Specialized Type II Hospitals
1st	1	11	0	7	3
2nd	6	9	2	2	2
3rd	0	14	0	1	0
4th	1	10	2	0	1
5th	2	10	1	0	0
6th	6	19	2	1	0
7th	3	4	1	0	0

**Table 2:** Pairwise correlations of the independent variables.

	Beds	Inpatients	Days of Stay	Outpatients	Hemodialysis	Surgeries	Laboratory Tests	Employees	Medical Imaging Tests	Inpatients 2	Days of Stay2	Scheduled Surgeries	Urgent Surgeries
Beds	1.00												
Inpatients	0.80	1.00											
Days of stay	0.91	0.90	1.00										
Outpatients	0.76	0.85	0.78	1.00									
Hemodialysis	0.31	0.39	0.31	0.50	1.00								
Surgeries	0.70	0.81	0.74	0.86	0.41	1.00							
Laboratory Tests	0.79	0.88	0.88	0.83	0.36	0.77	1.00						
Employees	0.92	0.88	0.92	0.85	0.35	0.80	0.92	1.00					
Medical Imaging Tests	0.63	0.71	0.68	0.79	0.39	0.78	0.72	0.74	1.00				
Inpatients 2 *	0.49	0.62	0.61	0.50	0.17	0.44	0.59	0.57	0.38	1.00			
Days of stay2	0.58	0.66	0.69	0.55	0.19	0.45	0.65	0.65	0.45	0.74	1.00		
Scheduled Surgeries	0.65	0.76	0.69	0.80	0.37	0.97	0.73	0.75	0.72	0.39	0.40	1.00	
Urgent Surgeries	0.61	0.60	0.59	0.69	0.39	0.78	0.61	0.67	0.72	0.29	0.38	0.68	1.00

* Inpatients2 is the number of patients in special units, and Days2 is their hospitalization days.

**Table 3:** Analytical results from the regression analysis of the total hospital operating costs (Logarithm of Total Costs (LogAllCosts)).

OLS Linear Regression Model		R² = 0.9519	Random Effect Model (xtreg, re)		R² = 0.9508
		Number of obs = 242			Number of obs = 242
		Coef. (Std.Err.)			Coef. (Std.Err.)
Constant	β_0_	12.024 (0.2135)	Constant	β_0_	12.182 (0.3094)
Number of Beds	β_1_	0.00172 (0.0073)	Number of Beds	β_1_	0.0273 (0.0047)
Hospital Type			Hospital Type		
General Hospital	β_2_	2.2382 (0.2176)	General Hospital	β_2_	1.9234 (0.3202)
University Hospital		3.8704 (0.4453)	University Hospital		1.8466 (0.6688)
Specialized Hospital I		2.3389 (0.3078)	Specialized Hospital I		1.9363 (0.4114)
Specialized Hospital II		4.0937 (0.3372)	Specialized Hospital II		3.96650 (0.3412)
Hospital Type # Beds			Hospital Type # Beds		
General Hospital	β_3_	0.00007 (0.00735)	General Hospital	β_3_	−0.02462 (0.0046)
University Hospital		−0.00439 (0.0073)	University Hospital		−0.02476 (0.0047)
Specialized Hospital I		−0.00105 (0.0073)	Specialized Hospital I		−0.02485 (0.0047)
Specialized Hospital II		−0.00499 (0.0087)	Specialized Hospital II		−0.02832 (0.0047)
Beds # Days	β_4_	−1.45 × 10^−8^ (2.30 × 10^−9^)	Beds # Days	β_4_	−1.45 × 10^−8^ (2.30 × 10^−9^)
Intensive Care Unit	β_5_	0.33931 (0.0883)	Intensive Care Unit	β_5_	0.20655 (0.0708)
Internal Patients	β_6_	0.0000241 (4.10 × 10^−6^)	Internal Patients	β_6_	0.0000184 (2.86 × 10^−6^)
Days of Stay	β_7_	0.0001878 (0.00005)	Days of Stay	β_7_	2.69 × 10^−6^ (1.62 × 10^−6^)
Employees	β_8_	0.001168 (0.0002)	Employees	β_8_	0.00065 (0.0002)
Location Island	β_9_	0.203897 (0.0819)	Laboratory Tests	β_9_	2.27 × 10^−7^ (6.60 × 10^−8^)
Hospital Type # Days					
General Hospital	β_10_	−0.00019 (0.00005)			
University Hospital		−0.00018 (0.00005)			
Specialized Hospital I		−0.000191 (0.00005)			
Specialized Hospital II		−0.0001858 (0.00005)			

Note that the symbol (#) represents the interaction of the two variables. *p*-values < 0.05.

**Table 4:** Akaike’s information criterion and Bayesian information criterion.

Model	Obs	ll (mul)	ll (model)	df	AIC	BIC
OLS Linear’s Regression model	121	−430.41	−63.2658	19	164.531	217.651
Random Effect Model	121	-	49.33912	18	−62.678	−12.354

Note: N = 121 used in calculating BIC.

## Data Availability

The datasets used and/or analyzed during the present study are available from the corresponding author on reasonable request.
